# Effects of plant diversity and abiotic factors on the multifunctionality of an arid desert ecosystem

**DOI:** 10.1371/journal.pone.0266320

**Published:** 2022-06-10

**Authors:** Yulin Shu, Lamei Jiang, Feiyi Liu, Guanghui Lv

**Affiliations:** 1 College of Ecology and Environment, Xinjiang University, Urumqi, Xinjiang, China; 2 Key Laboratory of Oasis Ecology of Education Ministry, Xinjiang University, Urumqi, PR China; Feroze Gandhi Degree College, INDIA

## Abstract

Many studies suggest that species diversity and abiotic factors promote ecosystem multifunctionality. However, whether ecosystem multifunctionality is impacted by phylogenetic diversity remains controversial. The present study tested this in an arid desert ecosystem in Ebinur Lake Basin using soil C:N ratio, soil pH, and soil salinity as abiotic factors, and species diversity and phylogenetic diversity as indicators of plant diversity. The effects of plant diversity and abiotic factors on single ecosystem functions (nutrient cycling, carbon stocks, water regulation, and wood production) and ecosystem multifunctionality were studied. We used structural equation modeling to assess the relationships among different functional groups and factors. The results showed that: (1) abiotic factors, particularly pH and C:N ratio in soil, had the strongest positive impact on multifunctionality (*P* < 0.001). The phylogenetic diversity and species diversity showed inconsistent changes, and their contribution to multifunctionality were not outstanding. (2) Abiotic factors were closely related to different ecosystem functions. Soil C:N had a significant positive effect on carbon stocks (*P* < 0.001), with an effect index of 0.89. Soil pH significantly enhanced nutrient cycling and water regulation. The role of plant diversity varied with the combination of different ecosystem functions. Phylogenetic diversity and species diversity influenced wood production, but showed opposite functions. (3) The importance of four single-ecosystem functions in an arid region was ranked as follows: carbon stocks > water regulation > nutrient cycling > wood production, emphasizing the importance of carbon elements in these ecosystems. These results improve our understanding of the drivers of multifunctionality in arid ecosystems, facilitating the elucidation of the influence of abiotic factors and phylogenetic diversity.

## Introduction

Biodiversity has sharply decreased with human destruction of the environment, with the resulting biodiversity loss leading to a decline in ecosystem functions and services [1]. This necessitates an exploration of the relationship between biodiversity and ecosystem functions.

Over the past 20 years, the relationship between biodiversity and ecosystem functions has generally focused on single ecosystem function and biodiversity. However, scientists have gradually discovered that ecosystems can perform multiple functions at the same time, and these can be affected by other factors such as biodiversity and abiotic factors [2]. Hector and Bagchi (2007) first proposed that biodiversity could have multiple impacts on ecosystems and found that maintaining more ecosystem functions requires a larger number of species than maintaining a single ecosystem function [3]. The study of biodiversity-ecosystem function relationships has shifted from exploring the role of a single ecosystem function to investigating the importance of an ecosystem’s capacity to sustain multiple ecosystem functions and services simultaneously, i.e., ecosystem multifunctionality [4]. Studies have ranged from small-scale and single ecosystem functional analyses to larger-scale and multispecies analyses of ecosystems and ecosystem functions [5]. The combination of different aspects of biodiversity with single functions and multifunctionality are essential to ecosystem conservation and the effective use thereof. The synergism of single functions are largely considered when studying the relationship between biodiversity and multiple functions as single functions such as nutrient cycling, carbon stocks, and primary production promote multifunctionality [6]. Some researchers have proposed that water is the most important limiting factor affecting arid desert plants in an ecosystem. Ignoring the trade-offs or synergies between these functions will result in erroneous estimates of overall ecosystem function measures [7].

Plant diversity includes three dimensions: species diversity, functional diversity, and phylogenetic diversity. All three dimensions are related to ecosystem functions, and both species diversity and functional diversity are known to play significant roles in ecosystem multifunctionality [8]. However, current understanding of the impact of phylogenetic diversity on ecosystem multifunctionality is limited. Phylogenetic diversity refers to the sum of species phylogenetic distances in a community and is influenced by the average interspecific genetic relationship and the number of species in the community [9]. Some studies have found that closer genetic relationships and higher phylogenetic diversity in plants can reduce competition, thus improving ecosystem functioning [10]. In some special circumstances, especially in arid areas, the positive effects of species diversity on ecosystem functioning under drought disturbances are weakened [11]. Phylogenetic diversity considers the hidden traits related to dryland functions, and dryland multifunctionality increases with the diversity of an evolutionary lineage [12]. The study of phylogenetic diversity can analyze the status and cause of a community’s species composition, as well as explore the ecological processes of species’ coexistence in a community [13, 14]. These findings suggest that phylogenetic diversity might be the best explanatory biodiversity index for multifunctionality.

The effect of abiotic factors on ecosystem functioning is also significant. Differences in abiotic factors result in a variety of conditions and nutritional supply levels for the growth of various plants, and soil abiotic factors are particularly important, as these are the basis of plant growth. Of the 17 essential elements needed for plant growth, 14 are obtained from soil [15]. As different abiotic factors have different effects on biodiversity and ecosystem functions, pathways in affecting ecosystem multifunctionality differ. In China, for example, it is suggested that soil pH may be an important driver of ecosystem multifunctionality and mediate the effects of precipitation on the ecosystem [2]. The C:N ratio is the most important environmental predictor of ecosystem multifunctionality during forest secondary succession [16]. At the same time, whether the response of abiotic factors to ecosystem multifunctionality is consistent with its response to single ecosystem function has also become pertinent. The improvement of one factor is likely to amplify the combination of ecosystem single functions and the role of ecosystem multifunctionality [17, 18]. Therefore, it is of great ecological significance to explore the influence of abiotic factors on single functions and multifunctionality and whether responses to these factors are consistent.

Desert ecosystems in arid areas are exposed to environmental stresses such as drought, salinization, and infertility for long periods of time. Different combinations of biodiversity, abiotic factors, and single ecosystem functions may all have their own characteristic responses to ecosystem multifunctionality. Taking the arid desert ecosystem of the Lake Ebinur Basin as the research subject, species and phylogenetic diversity were selected to represent plant diversity, and the soil C:N ratio, soil pH, and soil salinity were selected as abiotic factors. Regression analysis and structural equation models were used to explore the effects of biotic factors (plant diversity) and abiotic factors on water regulation, carbon stocks, wood production, and nutrient cycling for single functions and their multifunctionality in arid desert ecosystems. This was done to provide a standard for biodiversity conservation and management in arid areas as well as to answer the following scientific questions:

1. What are the effects of species diversity and phylogenetic diversity on particular ecosystem functions and on ecosystem multifunctionality? Which dimension of diversity plays a larger role?
2. How the two types of factors (diversity and abiotic) jointly contribute to ecosystem multifunctionality and through what pathways?

## Materials and methods

### Overview of the research area

The study area is located in Ebinur Lake Wetland National Nature Reserve (44°30′–45°09′N, 82°36′–83°50′E). It is also the center of the Ebinur Basin. The region has a dry typical continental climate with an annual precipitation of only about 89.80–169.70 mm and strong potential annual evaporation is higher than precipitation, which ranges from 1,569–3,421 mm. The mean annual temperature is 8°C. The reserve areas are low-lying natural saline-alkali basins where main water recharge and salt-rich sediments occur in Xinjiang [19]. The soil parent material is sandy soil, grey-brown desert soil, and grey desert soil, which are severely affected by desertification. The study area is predominated by psammophytic vegetation, mesozoic vegetation, and halophytic vegetation. The main plants are *Populus euphratica*, *Haloxylon ammodendron*, *Halimodendron halodendron*, *Alhagi sparsifolia*, *Reaumuria soongarica*, *Nitraria roborowskii*, *Apocynum venetum*, *Phragmites australis*, *Halocnemum strobilaceum*, *Salsola collina*, and *Suaeda glauca* [20]. Due to human damage and rapid land-use changes, the vegetation in the Ebinur Basin has become scarce and the diversity has substantially deteriorated [21].

### Sample setting and plant community survey

**Fig 1:**
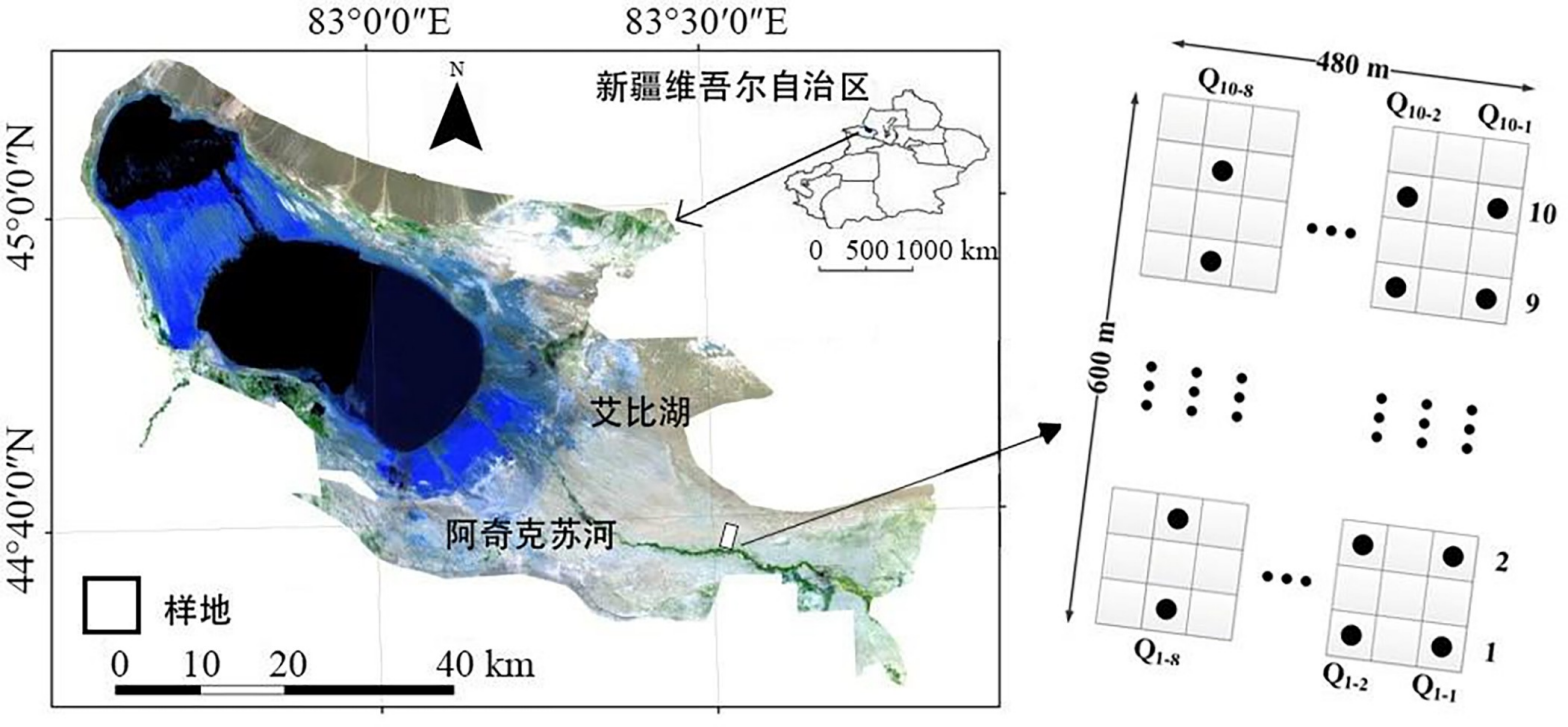
The study area and location of the plots. Autonomous Region is downloaded from The Gateway to Astronaut Photography of Earth website (https://eol.jsc.nasa.gov/SearchPhotos/). Image courtesy of the Earth Science and Remote Sensing Unit, NASA Johnson Space Center. Because the map downloaded from this website is free and open to scholars, our study does not need to supply a copyright notice.

Within the arid desert area of the Ebinur Lake National Wetland Nature Reserve, which is represented by Ebinur Lake in the following article, perpendicular to the Achiksu River, a natural water and salt gradient basin were formed along the soil moisture and salt gradient from far and near the river. Within an east-west distance of 480 m and a north-south distance of 600 m, from Q1-1 (plot number), a total of 80 plots, which measured 30 m × 30 m each, were taken at a sampling location of 30 m (see Fig 1). The names of all species in the plots were recorded and their height, coverage, and abundance were measured. A GPS was used to record the latitude, longitude, and altitude of the plots.

### Physicochemical properties of the soil

Soil samples were collected by diagonal sampling in each 30 m × 30 m sample, and each was sampled at depths of 0–10 cm, 10–20 cm, and 20–30 cm. Three layers of soil samples were collected in an aluminum box that had been weighed in advance. After collection, the number of samples was recorded, and the samples were transported to the laboratory, fresh soil weight was measured with a precision balance (1/10,000), and quality was noted. In the laboratory, the soil was dried and weighed for later soil quality and moisture calculations. Moreover, another soil sample was collected into a self-sealing bag. After air-drying, the sample was ground and sifted. The determination method of soil factors is shown in Table 1.

**Table 1:** Indicators of soil factors.

Determination of soil factors	Method	Role instruments
Soil water content (SWC, %)	Drying weighing method	Oven; 1/10,000 balance
Soil content (SSC, ms/cm)	Residue drying method	Oven; 1/10,000 balance
Soil organic carbon content (SOC, g/kg)	Potassium dichromate heating method	Iron platform
Soil total phosphorus content (TP, g/kg)	HClO_4_^-^H_2_SO_4_^-^ molybdenum antimony resistance colorimetric method	Spectrophotometer
Soil available phosphorus content (AP, mg/kg)	0.5 Mol NaHCO_3_ anticolorimetric method for extracting molybdenum and antimony	Reciprocating oscillator; spectrophotometer
Soil total nitrogen content (TN, g/kg)	Kjeldahl determination method	Cooker; Kjeldahl nitrogen-fixing apparatus; drop tube
Soil ammonium nitrogen content (AN, mg/kg)	2 mol·L ^−1^ KCl extract-indophenol blue colorimetry	Reciprocating oscillator; spectrophotometer
Soil pH	Glass electrode method	Total pH total

## Data analysis

### Species diversity calculation

The species richness index, Shannon-Wiener index, and Simpson index were selected for species diversity calculations.

Species richness index:

D=S,

Shannon-Wiener index H′:

$$H^{\prime }=‐\overset{S}{\underset{i=1}{\sum }}P_{i}\ln P_{i},\;and$$

Simpson index F:

$$F=1‐\overset{S}{\underset{i=1}{\sum }}P_{i}^{2},$$

where H′ is the Shannon-Wiener diversity index, F is the Simpson dominance index, S is the number of species in the sample, and P_i_ is a ratio of the number of individuals of the i^th^ species to the total number of individuals.

### Phylogenetic reconstruction of species in wooded plots

**Fig 2:**
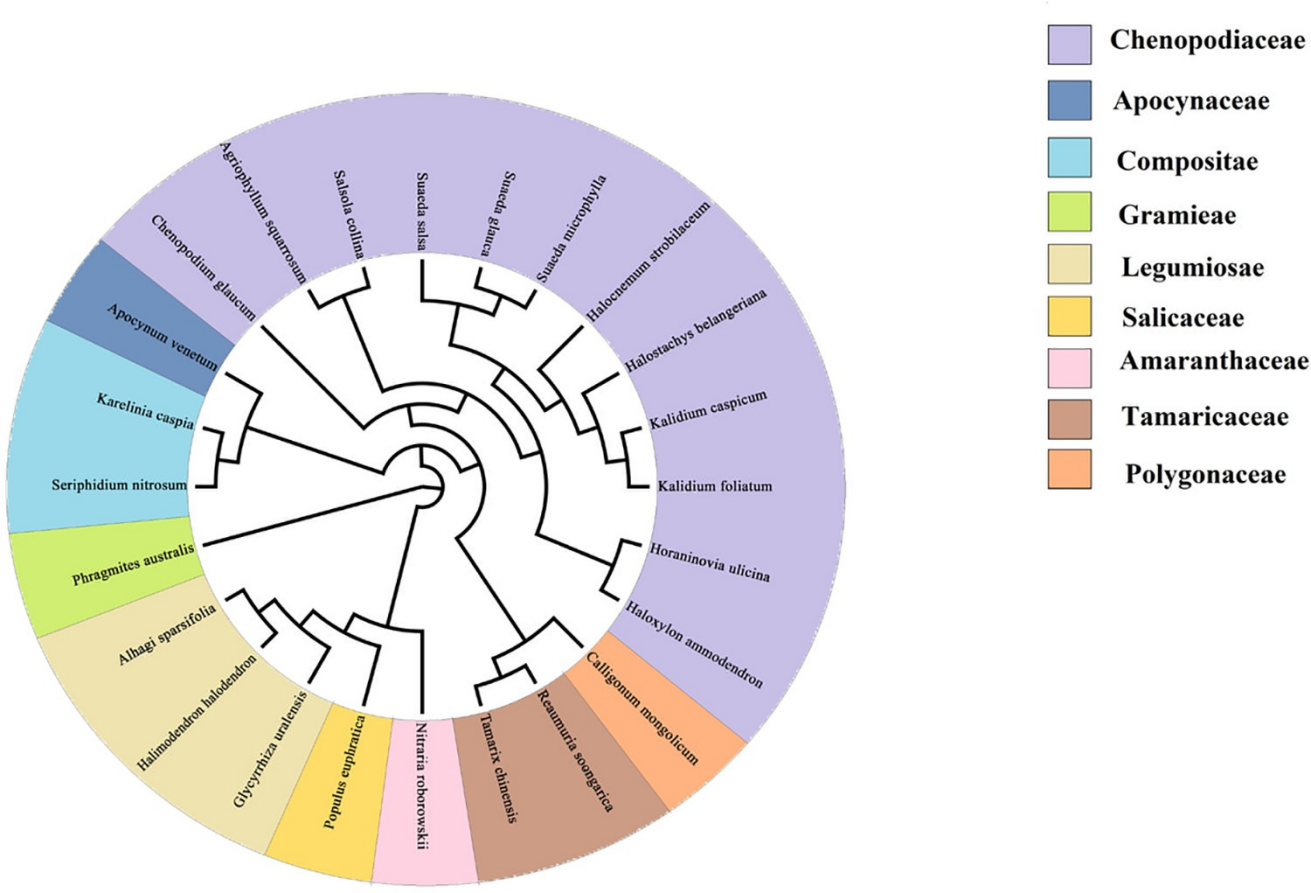
Plant phylogenetic tree of the sample plot.

The species and genus information obtained from the samples were submitted to the plant phylogenetic database software Phylomatic [21]. Based on the APGb system, an evolutionary tree was generated online using the phylogeny described by Zanne et al. (2014) [22] and was mapped with Figtree. The phylogenetic tree is shown below (Fig 2).

### Phylogenetic diversity (PD) calculations

PD is the sum of all branch lengths in the phylogenetic wooded plots of species in the community. The net relatedness index (NRI) and nearest taxon index (NTI) represent the phylogenetic α diversity of the community. The formulae are as follows:

$${\mathrm{NRI}}_{\mathrm{sample}}=‐1\times \frac{{\mathrm{MPD}}_{\mathrm{sample}}‐{\mathrm{MPD}}_{\mathrm{randsample}}}{\mathrm{SD}({\mathrm{MPD}}_{\mathrm{randsample}})},\;and$$

$${\mathrm{NTI}}_{\mathrm{sample}}=‐1\times \frac{{\mathrm{MNTD}}_{\mathrm{sample}}‐{\mathrm{MNTD}}_{\mathrm{randsample}}}{\mathrm{SD}({\mathrm{MNTD}}_{\mathrm{randsample}})},$$

where MPD and MNTD are the average evolutionary distance between species and the average evolutionary distance between the nearest species, respectively, and MPD_sample_ and MNTD_sample_ are the respective actual observations; MPD_randsample_ and MNTD_randsample_ are the respective average values calculated from the results of randomizing 999 times on the phylogenetic tree; and SD is the standard deviation.

### Calculation of a single function of an ecosystem

In this study, nutrient cycling, carbon stock, water regulation, and wood production were selected as ecosystem services at the plot level to evaluate the single functions of the ecosystem. Soil total phosphorus content (TP), soil total nitrogen content (TN), soil available phosphorus content (AP), and soil ammonium nitrogen content (AN) were used to characterize nutrient cycling; soil organic carbon content (SOC) was used to characterize carbon stocks; soil water content was used to characterize water regulation; and community-level weight means (CWM) was used to calculate the plant height weighted average of the community to characterize wood production [16]. The formula is as follows:

$$\mathrm{CWM}=\overset{S}{\underset{i=1}{\sum }}P_{i}\times {\mathrm{trait}}_{i},$$

where P_i_ represents the relative abundance for the i^th^ species; and trait_i_ represents the species i trait value.

### Ecosystem multifunctionality calculations

Ecosystem nutrient cycling, carbon stock, water regulation, and wood production in this study used the means to convert the values for the determination of ecosystem function in the plots. Then, these functional indicators were standardized using the Z worth fraction method. The mean value of each functional value was calculated as a multifunctionality index. The formula is as follows:

$${\mathrm{MF}}_{a}=\frac{1}{F}\overset{F}{\underset{i=1}{\sum }}g(r_{i}(f_{i})),$$

where MF_a_ represents the multifunctionality of the ecosystem, F represents the number of functions measured, f_i_ represents the measured value of functional i, r_i_ is a mathematical function that transforms f_i_ into positive values, and g represents the standardization of all measurements.

### Statistical analysis

PD, comdist, and comtrust in the PhyloCom software were used to obtain the NTI and NRI of phylogenetic richness. Regression analysis was used to investigate the relationship among abiotic factors, species diversity, and phylogenetic diversity and their relationship with ecosystem multifunctionality. Regression analysis was performed using SPSS24.0. The calculation of community weighted average and random forest analysis were conducted using R4.0.2 (www.r-project.org), and the construction of the structural equation model was used for the correlations between different dimension diversity, abiotic indices, and multiple functions, which were performed using AMOS24.0.0.

## Results

### Relationship between plant diversity, abiotic factors, and ecosystem multifunctionality

Species diversity and phylogenetic diversity were different dimensions of plant diversity, soil pH, soil salinity, and the soil C:N ratio as abiotic factors to explore the relationship among the five factors and ecosystem multifunctionality in an arid desert area. The final models featured different relationships between predictors and the response for the most significant results.

**Fig 3:**
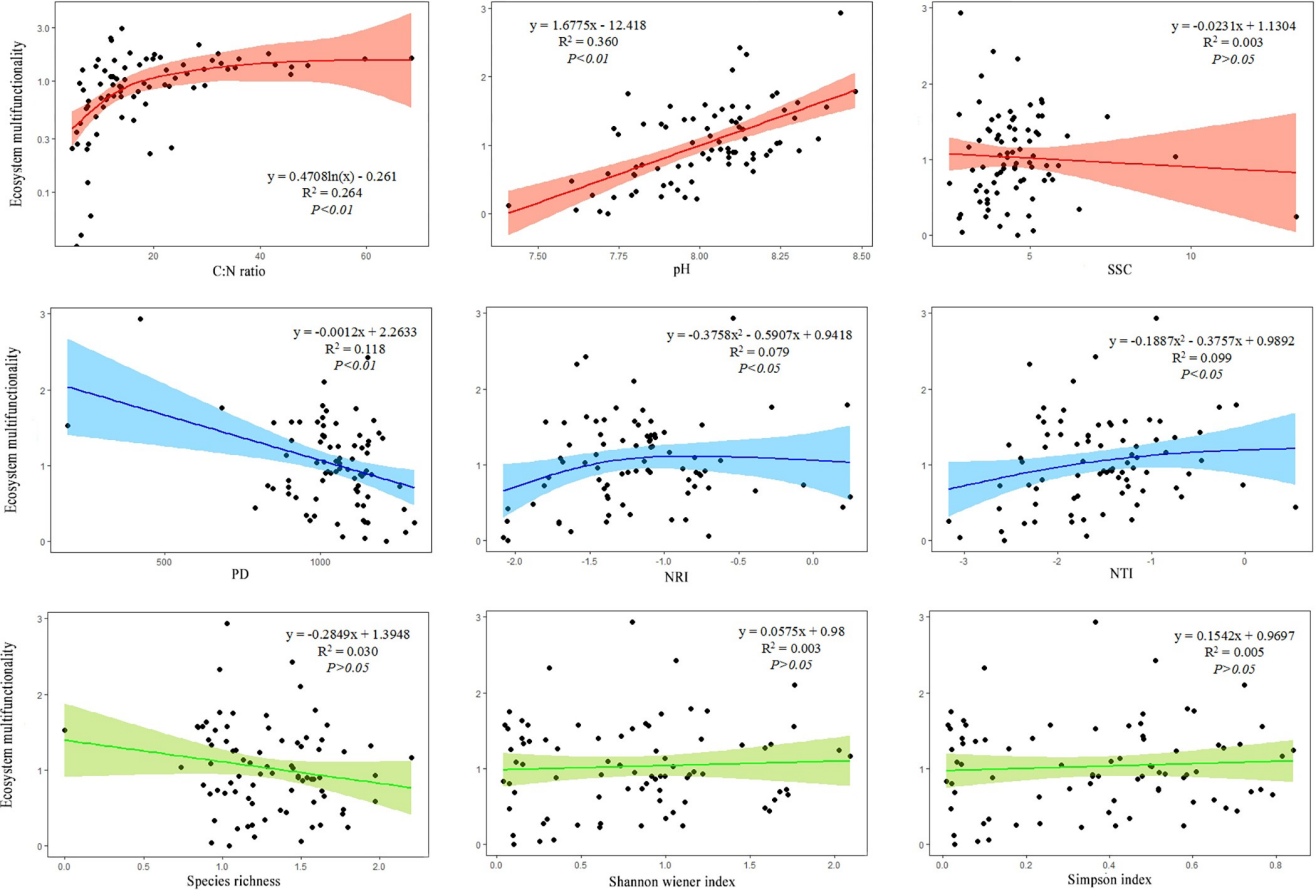
Relationship among plant diversity, abiotic factors, and ecosystem multifunctionality.

As shown in Fig 3, the soil C:N ratio, soil pH, and PD were significantly associated with ecosystem multifunctionality (*P* < 0.01) (Fig 3A, 3B and 3D); NTI and NRI showed a significant negative single peak curve for ecosystem multifunctionality (*P* < 0.05), while species richness, Shannon-Wiener index, Simpson diversity index, and soil salinity were not significantly associated with ecosystem multifunctionality (*P* > 0.05) (Fig 3A, 3B, 3D–3F). The soil C:N ratio and soil pH well explained ecosystem multifunctionality (R^2^ = 0.264, R^2^ = 0.360), while the other indices had little effect on ecosystem multifunctionality. Overall, the correlation with abiotic factors was greater than with plant diversity, suggesting the role of abiotic factors in maintaining ecosystem multifunctionality.

### Effects of plant diversity and abiotic factors on single ecosystem function

#### Effects of plant diversity and abiotic factors on nutrient cycling function

Research has shown that plant diversity and abiotic factors are closely related to ecosystem function. The relationship between plant diversity and single function depends not only on the function of species but also on the influence of abiotic factors [23]. Therefore, it is necessary to consider plant diversity and abiotic factors as multiple variables to estimate the response to the single function of an ecosystem. In this study, the four single functions of nutrient cycling, carbon stocks, water regulation, and wood production were selected, and the role of plant diversity and abiotic factors was explored using a structural equation model.

**Fig 4:**
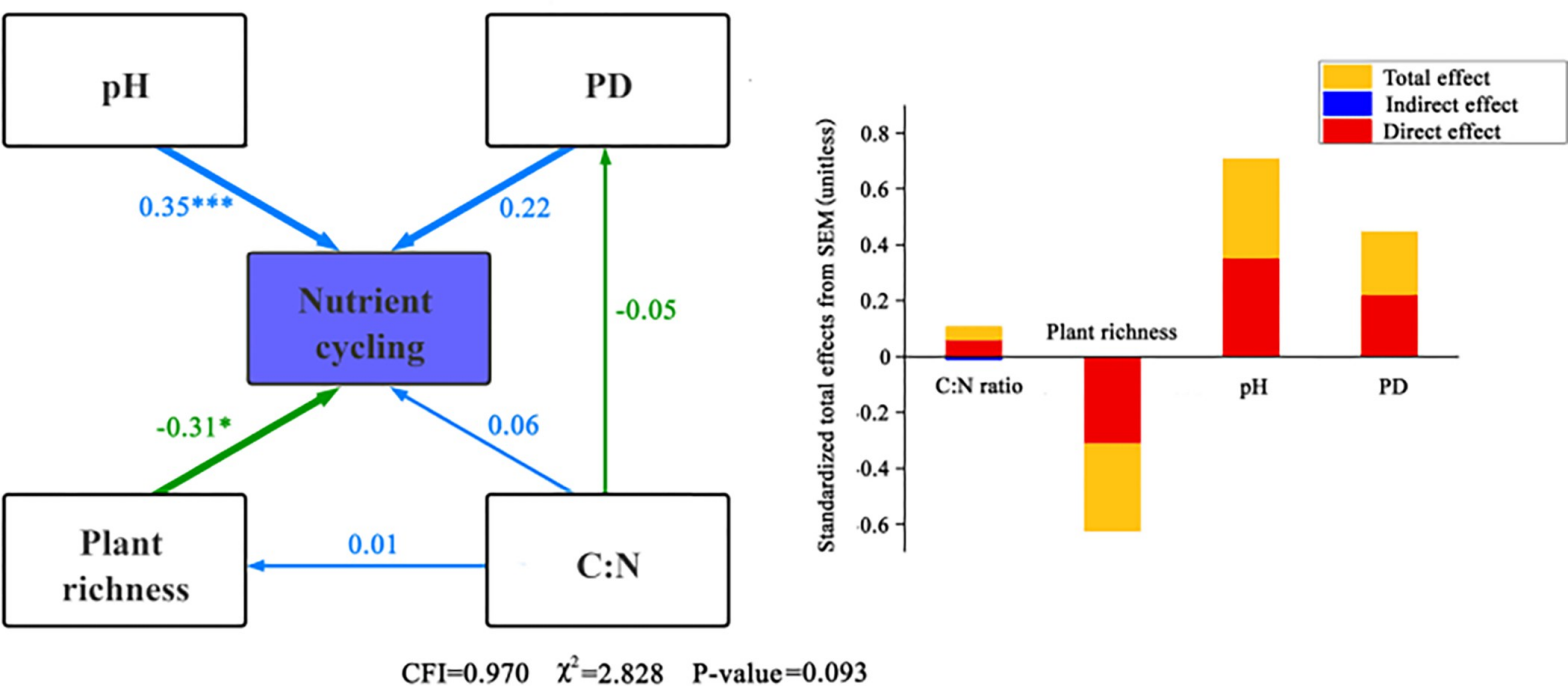
A structural equation model of the relationship between plant diversity, abiotic factors, and nutrient cycling. The blue and green arrows indicate positive and negative relationships, respectively. The width and strength of the arrow are proportional. The number near the arrow is the normalized path coefficient, reflecting the magnitude of the effect of causality. The significance level is as follows: * *P* < 0.05, * *P* < 0.001, the same as below.

The effects of plant diversity and abiotic factors on nutrient cycling function are shown in Fig 4. To ensure that the whole fitting effect of the model met the requirements, the soil salinity, phylogenetic diversity NTI and NRI, and Shannon-Wiener and Simpson diversity indices with low path coefficients in the nutrient cycling models were removed. A structural equation model of species richness, PD, soil pH, and the soil C:N ratio was obtained (Model NC = 2.828, CFI = 0.970) and indicated that the results fitted well. Nutrient cycling was most affected by soil pH (*P* < 0.001), with a path coefficient of 0.35. PD and the soil C:N ratio did not directly affect nutrient cycling (*P* > 0.05), whereas the soil C:N ratio indirectly affected nutrient cycling by exerting a negative influence on species richness, with an indirect effect coefficient of −0.012. Species richness had a significant negative effect on nutrient cycling, with a path coefficient of −0.31(*P* < 0.01).

#### Effects of plant diversity and abiotic factors on carbon stock function

**Fig 5:**
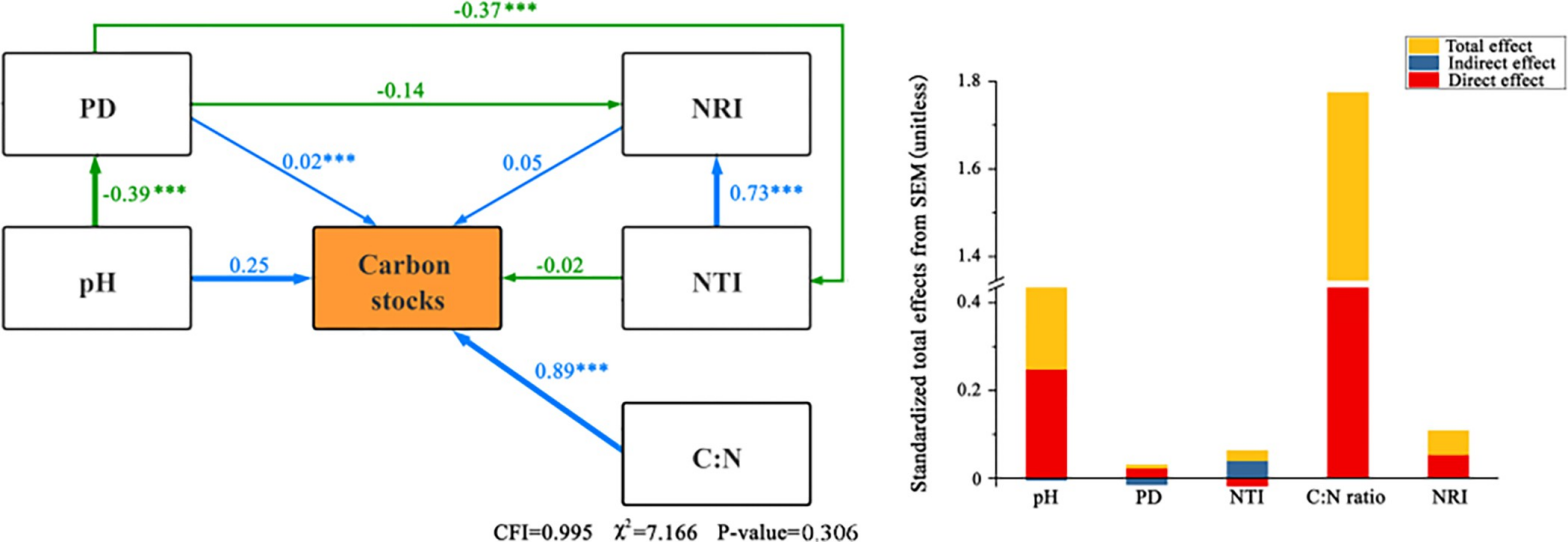
Structural equation model of the relationship between plant diversity and abiotic factors and carbon stocks.

The structural equation model comprising phylogenetic diversity, soil pH, and the soil C:N ratio with carbon stocks is shown in Fig 5, indicating an overall good fitting effect of the model (NC = 1.194, CFI = 0.995). The ratio of soil C:N to carbon stocks had a significant positive effect (*P* < 0.001), and the direct effect was the largest, with a path coefficient of 0.89. PD also showed a significant positive correlation with carbon stocks (*P* < 0.001), but the influence level was low with a direct effect of 0.02. PD also played an indirect role in carbon reserves through NTI, and the indirect effect index was −0.015. The positive effect of soil pH and NRI on carbon stocks was not significant (*P* > 0.05), with path coefficients of 0.25 and 0.05. Only NTI had a direct negative effect on carbon stocks, but this was not significant (*P* > 0.05). NTI also played a positive role in carbon stocks by influencing NRI, but the effect was very low. PD, NTI, and NRI reflect plant diversity in the models, while the soil C:N ratio and soil pH reflect abiotic factors, and thus abiotic factors overall had a greater effect on carbon stocks than plant diversity.

#### Effects of plant diversity and abiotic factors on water regulation

**Fig 6:**
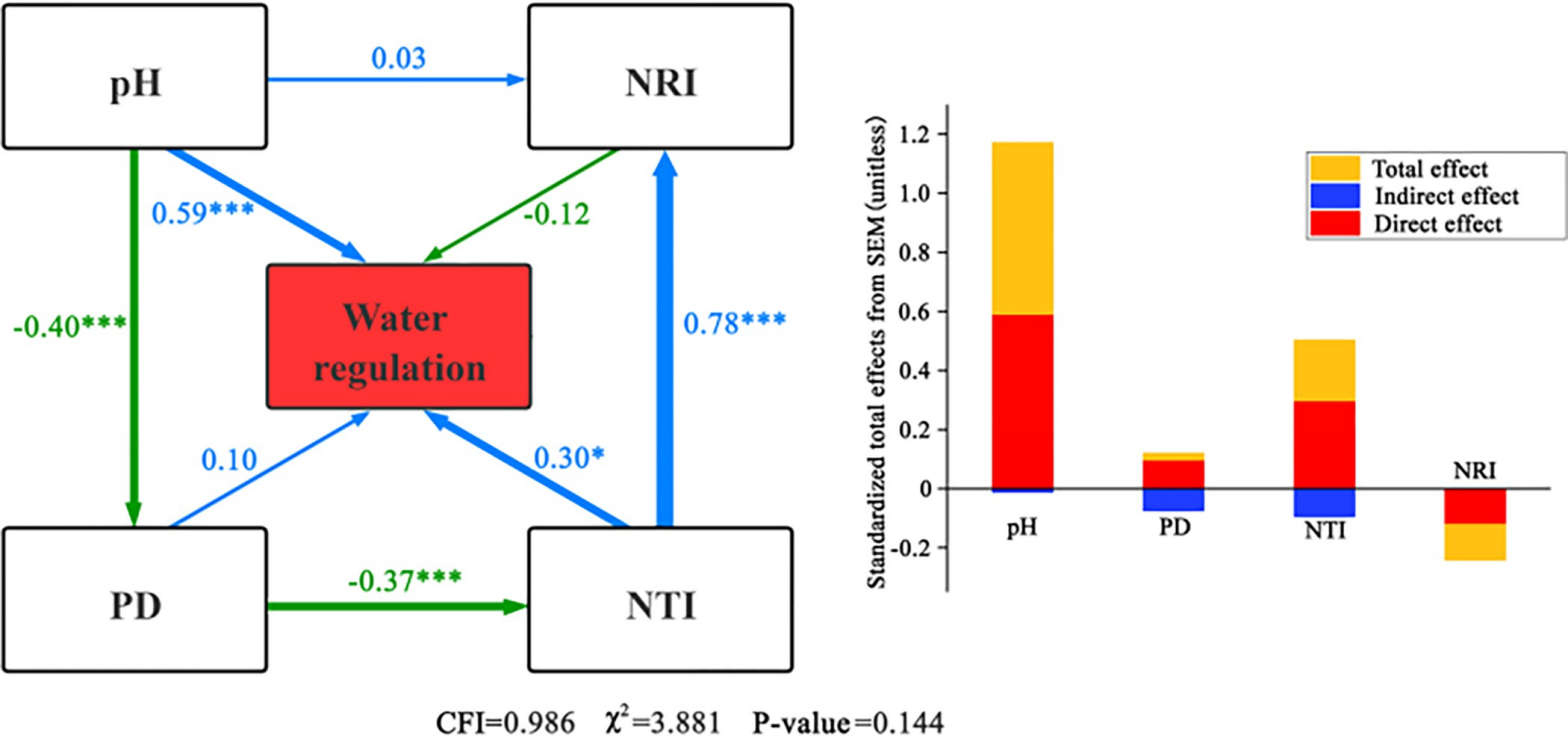
Structural equation model of the relationship between plant diversity, abiotic factors, and water regulation.

The structural equation model of the relationship between phylogenetic diversity, soil pH, and water regulation is shown in Fig 6 and indicates that the overall fitting of the model was good (Model NC = 1.941, CFI = 0.986). Increased PD, NTI, and soil pH were associated with increased water regulation. NTI and soil pH better explained water regulation, whereas NTI had a significant positive correlation with water regulation (*P* < 0.05) with a direct path coefficient of 0.30. Soil pH not only had a significant positive effect on water regulation (*P* < 0.001) but also played a leading role in the model, with a path coefficient of 0.59. Soil pH and NTI also indirectly acted on water regulation through their effects on NRI, but the indirect effects of soil pH and NTI were very small, with path coefficients of −0.012 and −0.075, respectively. Although PD had no significant effect on water regulation function (*P* > 0.05), it acted indirectly on water regulation via NTI. The NRI had a negative effect on water regulation (*P* > 0.05) but the effect was weak (path coefficient = −0.12). Although plant diversity had more factors than abiotic factors in the model, the abiotic factors still played a primary role in water regulation.

#### Effects of plant diversity and abiotic factors on wood production function

**Fig 7:**
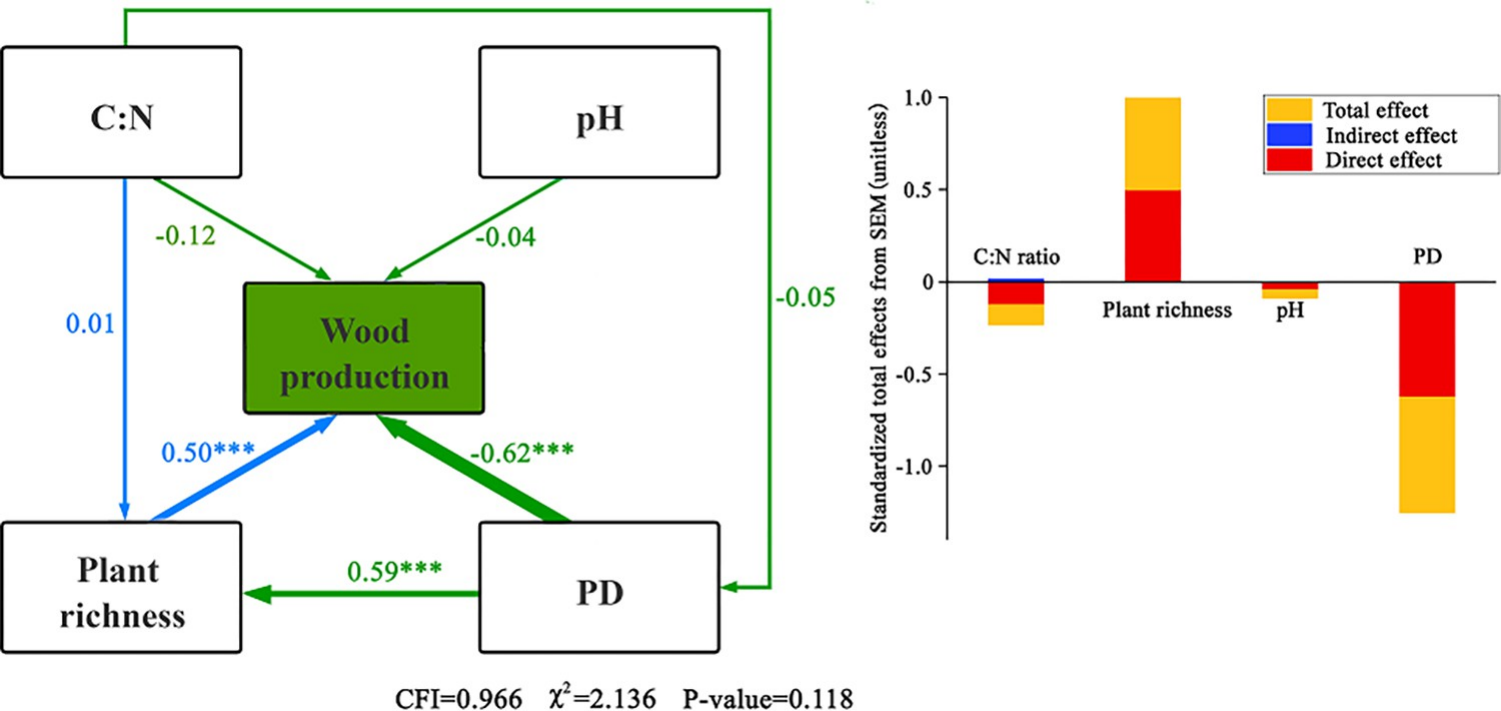
Structural equation model of the relationship between plant diversity, abiotic factors, and wood production.

The structural equation model of plant diversity, abiotic factors, and wood production is shown in Fig 7. PD was the most important factor affecting wood production (*P* < 0.001), with a path coefficient of −0.62. Species richness had a positive and direct effect on wood production (*P* < 0.001), with a path coefficient of 0.50. Although two diversity factors had opposite correlations with wood production, these were highly positive correlated (*P* < 0.001). Soil pH and soil C:N had no significant effect on wood production (*P* > 0.05). Soil C:N ratio also had a positive indirect effect on wood production through species richness, and although the effect was low (indirect effect = 0.015), these factors influenced wood production.

### Effects of plant diversity and abiotic factors on ecosystem multifunctionality

**Fig 8:**
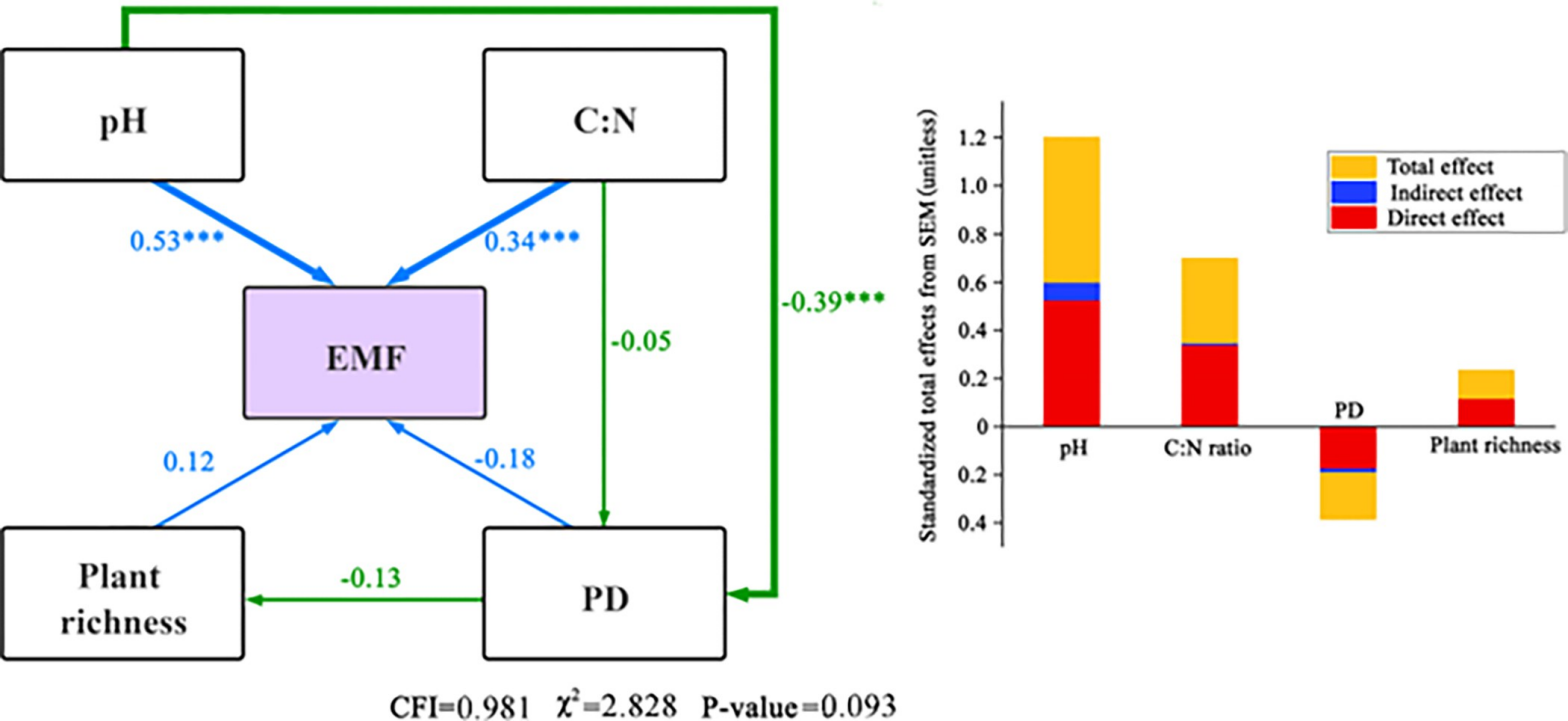
Structural equation model of plant diversity and abiotic factors with ecosystem multifunctionality.

Ecosystem multifunctionality can explain the comprehensive response of ecosystem functions [24], which is of great significance; however, little is known about the interactive effect of ecosystem multifunctionality and its driving factors in arid desert areas. Therefore, structural equations were used to explore the direct and indirect effects of plant diversity and abiotic factors on the maintenance of ecosystem multifunctionality and identify the main factors influencing ecosystem multifunctionality. As shown in Fig 8, the overall fitting effect of the model was good (NC = 2.828, CFI = 0.981). Soil pH was the most important factor affecting ecosystem multifunctionality and was positively correlated with ecosystem multifunctionality (*P* < 0.001), with a direct path coefficient = 0.59. The soil C:N ratio, also significantly positively correlated with ecosystem multifunctionality (*P* < 0.001), had lesser impact (path coefficient = 0.34). Soil pH and the C:N ratio not only played a direct role in ecosystem multifunctionality, but also had a negative indirect role in ecosystem multifunctionality by affecting PD.

### Importance of single ecosystem function in arid desert areas

**Fig 9:**
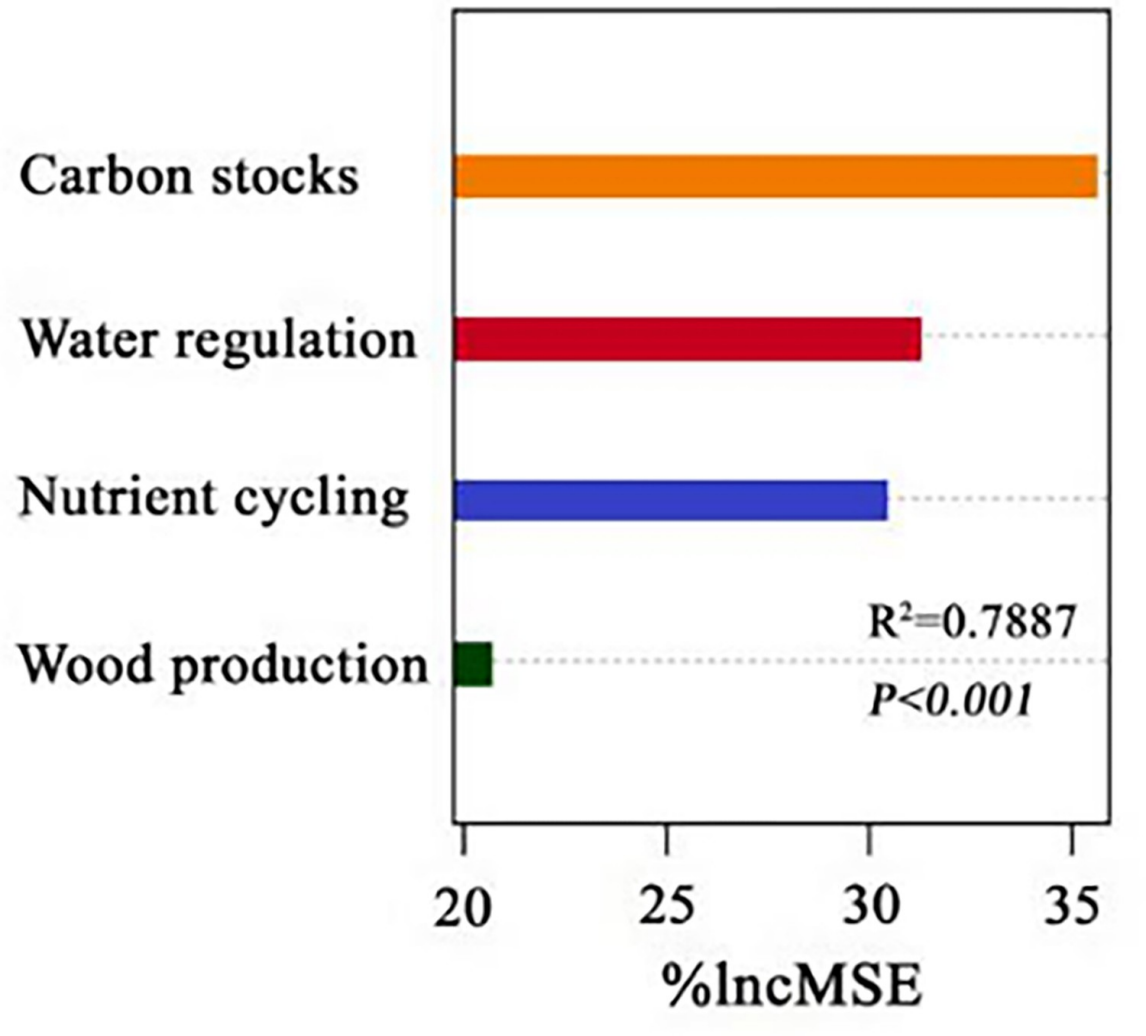
Functional importance ranking of single ecosystem function in arid desert areas.

To maximize ecosystem functions and services in arid desert areas and to meet the multifunctionality optimization of ecosystems, it is essential to investigate the importance of single ecosystem functions and determine which single ecosystem function plays a major role in arid desert areas. Understanding the predominant role of single ecosystem functions can promote the stable coexistence and species diversity of plants and maximize ecosystem multifunctionality. Therefore, in a random forest model, ecosystem nutrient cycling, carbon stocks, water regulation, and wood production function groups were input as four independent variables, and ecosystem multifunctionality as the dependent variable was used to explore the dominant functional factors in arid desert areas. As shown in Fig 9, in this study area, carbon stocks and water regulation exhibited the greatest feature importance, nutrient cycling was second, and wood production was last, and the proportion of each function could be weighed properly in order to maximize the management of multifunctionality. Carbon stocks are the main function factor affecting the arid desert area, mainly influencing ecosystem function.

## Discussion

### Effects of plant diversity and abiotic factors on ecosystem multifunctionality

In arid desert regions, we found that species diversity increased ecosystem multifunctionality, but did not significantly affect ecosystem multifunctionality, and that multifunctionality was better explained by abiotic factors. This may because species are the basic unit of the study, and it is difficult to distinguish the contribution of different species to biodiversity and multiple ecosystem functions [25]. Multifunctionality is an ecosystem ability that can simultaneously provide multiple functions and services [3]. In this study, we selected functions related to different biogeochemical cycles [26], so it is difficult to truly reflect the role of simple species diversity index in multifunctionality.

In the structural equation model, PD had no significant effect on ecosystem multifunctionality in the arid desert area, suggesting that phylogenetic diversity is a poor contributor to multifunctionality, which is consistent with the findings of a previous study [27]. Phylogenetic diversity plays only a minor role in ecosystem multifunctionality, which may be due to the fact that soil functional traits in arid desert areas are not conserved in phylogenies [28]. In the future, we should pay attention to the role of phylogenetic diversity in interpreting the mechanisms structuring communities.

In this study, biodiversity-ecosystem multifunctionality relationships were not obvious. This may have been because we did not provide more comprehensive attributes of biodiversity such as functional diversity and functional trait diversity [29]. Therefore, in future research, greater attention should be paid to different dimensions of the diversity effect on multifunctionality in arid desert areas.

The direct effects of the soil C:N ratio and soil pH on ecosystem multifunctionality were 0.34 and 0.53, respectively, suggesting that abiotic factors, especially soil factors, significantly affect ecosystem multifunctionality (Fig 8). This may be because an increased soil C:N ratio (without exceeding the threshold value) provides more nutrients for plant growth. As carbon can be decomposed by soil microorganisms and turned into inorganic matter for plant use, when the content of soil organic carbon and nitrogen increases, soil can promote plant growth and play an active role in ecosystem functioning [30]. During long-term natural selection, plants form their own specific requirements for soil pH, and soil pH affects the concentration of various ions in the soil solution, thus affecting the effectiveness of various elements on plants, which will greatly impact on soil fertility and plant growth. When the soil is alkaline, the availability of soil nutrients is high, thereby promoting metabolism and growth of plants in the ecosystem [31].

### Effects of plant diversity and abiotic factors on single ecosystem functions

The soil C:N ratio has a positive association with both nutrient cycling and carbon stock, and increasing soil C:N ratio enhances soil organic matter, which in turn may stimulate soil nutrients and soil carbon stocks [32]. Soil C:N has a significant positive effect on carbon stocks because a higher soil C:N ratio may more easily result in nutrient immobilization and limit the process rate, resulting in increases in carbon stocks in arid zone systems [16].

With the increase in soil pH, nutrient cycling and carbon stocks showed a significant increasing trend, which may be due to the fact that neutral to alkaline soil is essential for microorganisms such as bacteria and actinomycetes to survive. It is easy to release more nutrients and mineralize soil organic matter [33]. A positive correlation between soil pH and water was observed in this study, which is concordant to previous research results [34] that showed that soil pH plays an important role in the process of ecosystem water regulation. However, the specific relationship between water regulation and the pH ratio requires further investigation.

Species diversity has a significant positive effect on wood production, which may due to the fact that an increase in species diversity improves resource utilization efficiency of a community, as well as enhance community production and ecosystem function [35]. Therefore, when species diversity increases, it also promotes the relationship between species diversity and wood production. This result shows the important impact of species diversity on ecosystem function in the arid desert area. However, species diversity had a significant negative correlation with nutrient cycling, which is discordant to the findings of a previous study [36]. This may be due to the deep and developed plant roots in arid desert areas, which can transfer deep microelements to the topsoil layer and redistribute these through ecological processes such as litterfall and penetrating rain, to enter into nutrient cycles [37]. Most plants in arid desert areas are shrubs with high species diversity. As shrubs are adaptable and have a slow metabolism, there is less litterfall. The study area is located in an inland arid area with a dry climate and very low precipitation, and the precipitation almost completely evaporates before being absorbed by the soil [38]. Therefore, as the predominant species of arid areas, the species diversity of shrubs is not likely to play a positive role in nutrient cycling through litterfall and penetration. However, species diversity had no significant effect on water regulation and carbon stock function, which may be due to the uneven supply of resources in different plant communities in arid desert areas. The functional traits of different species vary greatly [39, 40]. Therefore, the effect of different functions on species richness is not consistent.

Phylogenetic diversity had a positive and significant effect on carbon stocks, which indicates that phylogenetic diversity is critical to maintain the C cycle. However, phylogenetic diversity had a significantly negative impact on wood production, which is discordant to previous findings that phylogenetic diversity does not play a significant role [41]. This might be attributed to the low genetic diversity of dominant species in the study area. Phylogenetic diversity had a significant impact on three single ecosystem functions: carbon stocks, water regulation, and wood production. However, the impact on ecosystem multifunctionality was not consistent, indicating that the role of phylogenetic diversity varies with the combination of different ecosystem functions.

### Impact of single ecosystem function importance in drylands

The random forest model showed that the importance values of the functions of four single ecosystems in the arid desert area were: carbon stocks > water regulation > nutrient cycling > wood production. Carbon stocks were the main functional factor affecting the multifunctionality of the arid desert area and played a major role in ecosystem multifunctionality. This suggests that carbon profoundly affects the processes of desert ecosystems in arid regions, possibly because carbon is an important determinant of plant growth and production. This is also consistent with previous studies that found that desert ecosystems are one of the biggest carbon sink regions in the world [42]. Water regulation function ranked second, indicating that water is an important functional factor in desert systems in the arid area of the Ebinur Lake Basin. Water is the main limiting factor for the growth of desert plants. Through root distribution, plants can redistribute the uneven distribution of water resources in the soil, thus promoting the water regulation of desert ecosystems in arid areas [43]. By occupying different proportions of a niche, different plants can comprehensively utilize potential water resources through coevolution and adaptation, promote the growth and diversification of plants, and promote the distribution and fixation of water in desert ecosystems [44]. Plants acquire nutrients during growth and retransmit them to the soil when the plant dies, which effectively accelerates the nutrient cycle rate of an ecosystem, while in arid desert systems, the nutrient cycling of plants is slow due to the low biomass and low species richness [45]. Thus, in an arid desert environment, nutrient cycling does not contribute significantly to ecosystem function. Finally, the contribution of tree production in the desert ecosystem was the lowest, which is likely due to the low coverage of shrubs and herbs in the arid area, as herbs contribute most of the production of desert ecosystems [46], and arbors with high crowns have less influence on tree productivity because of less quantity.

Our results also showed that ecosystem multifunctionality depends on different functional combinations, thereby reflects the high complexity of ecosystem functions [47]. Therefore, we should also consider the trade-off and synergy between functions, further enrich the research content and methods of diversity and multifunctionality, and promote the complementary utilization of resources in arid areas. This should significantly contribute to enhancing the capacity of ecosystem services in arid desert areas and to the future protection of biodiversity and ecosystem multifunctionality.

In addition, our study confirmed that the effects of environmental factors have a significant impact on multifunctionality and ecosystem functions, but the effect of biodiversity factors should not be ignored. We highlight the idea that we should add functional diversity to research in the future. Species, function and phylogenetic diversity are three dimensions of biodiversity [48], due to the contribution of the three dimensions of diversity to ecosystem function are different, so we suggest that species, function and phylogenetic diversity should be considered simultaneously in the future [6, 49]. Furthermore, the research on soil microbial diversity and multifunctionality also has become popular [50], soil microbial diversity play key roles in multifunctionality by participating in litter decomposition and nutrient mineralization [35]. And there is a strong interaction between plant, soil microbial diversity and multifunctionality [51]. Different soil microbial communities can also interact with aboveground plant communities to jointly affect multifunctionality at local and regional scales. In the future study, the correlation between soil microbial diversity and plant diversity, the impact of microbial diversity and plant diversity on ecosystem multifunctionality should be added in arid desert areas.

## Conclusions

This study analyzed the effects of different plant diversity and abiotic factors on multifunctionality in the Ebinur Lake Basin and found that soil C:N ratio and soil pH were the most important factors affecting single ecosystem function and ecosystem multifunctionality. Species diversity and PD had no significant impact on ecosystem multifunctionality. Assessment of the relative importance of nutrient cycling, carbon stocks, water regulation, and wood production indicated that ecosystem carbon stocks were the most important single ecosystem function affecting plant growth and development and multifunctionality in arid desert areas. We observed correlations between phylogenetic diversity, species diversity, and ecosystem multifunctionality, and plan to combine more attributes of biodiversity to multifunctionality in our subsequent studies.

## Supporting information

S1 Data (XLSX)
